# Supplementary material for: The Effects of Walnuts and Academic Stress on Mental Health, General Well-Being and the Gut Microbiota in a Sample of University Students: A Randomised Clinical Trial
Source: Nutrients. 2022 Nov 11;14(22):4776. doi: 10.3390/nu14224776 (PMC9697272; doi:10.3390/nu14224776)
Supplement: Supplementary file 1 [file nutrients-14-04776-s001.zip › nutrients-1988300-supplementary.pdf]

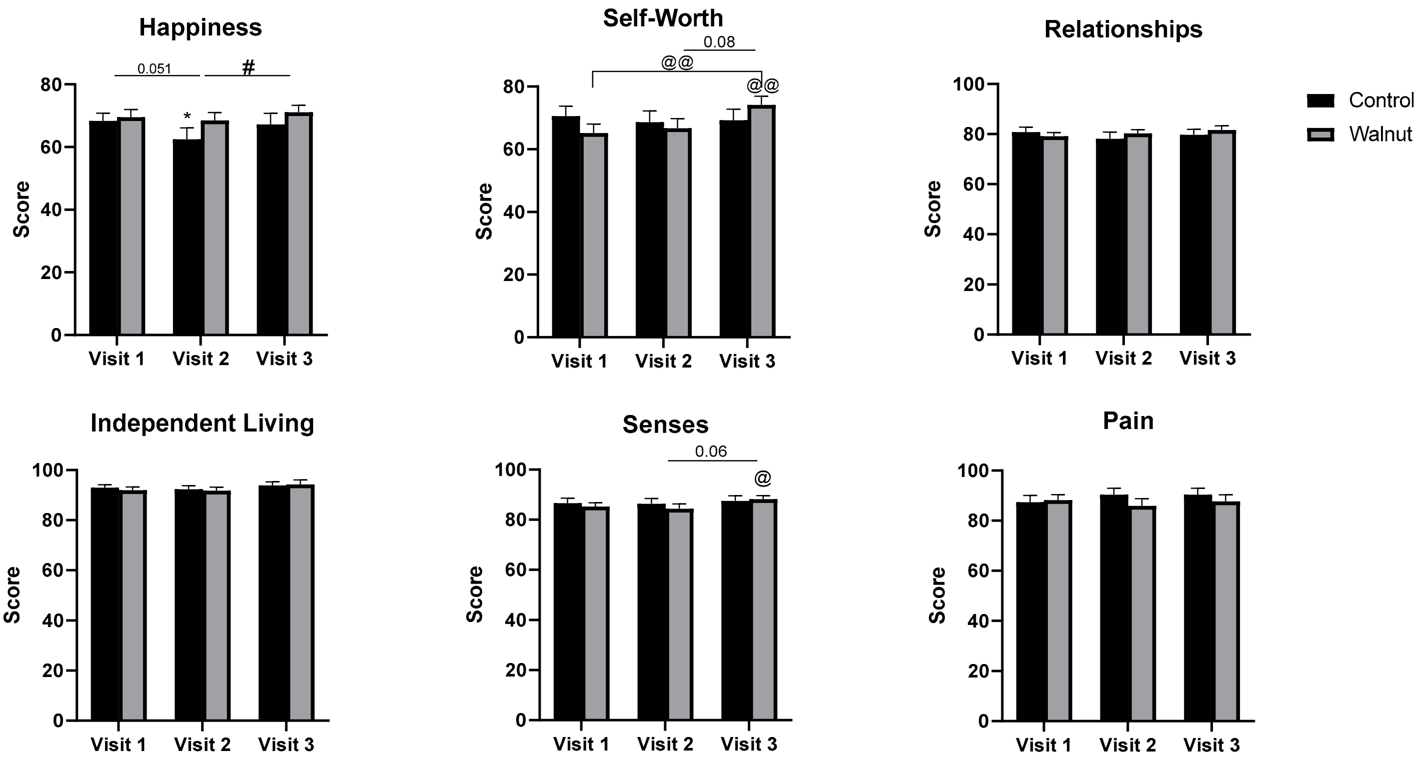

Figure S1. The effects of walnut consumption on perceived happiness, self-worth, relationships, independent living, senses and pain before, during and after the university examination period in the 8 dimension-Assessing Quality of Life Questionnaire. Clinical Visit 1 occurred during the first half of the university semester, Visit 2 occurred during the university examination period, and Visit 3 occurred at the end of the university semester. Mixed effects analyses followed by Bonferroni multiple comparisons. * Denotes a difference in control means compared to the immediately preceding visit unless otherwise indicated by lines, * = $p < 0.05$; @ denotes a difference in walnut means compared to the immediately preceding visit unless otherwise indicated by lines, @ = $p < 0.05$, @@ = $p < 0.01$; # denotes a difference in main effect of time (visit), # = $p < 0.05$. Visit 1: control n = 34; walnut n = 33. Visit 2: control n = 30; walnut n = 29. Visit 3: control n = 29; walnut n = 30. Small differences in sample size are due to participant withdrawals and incomplete responses to the questionnaire. Analyses performed on responses from all 3 cohorts.

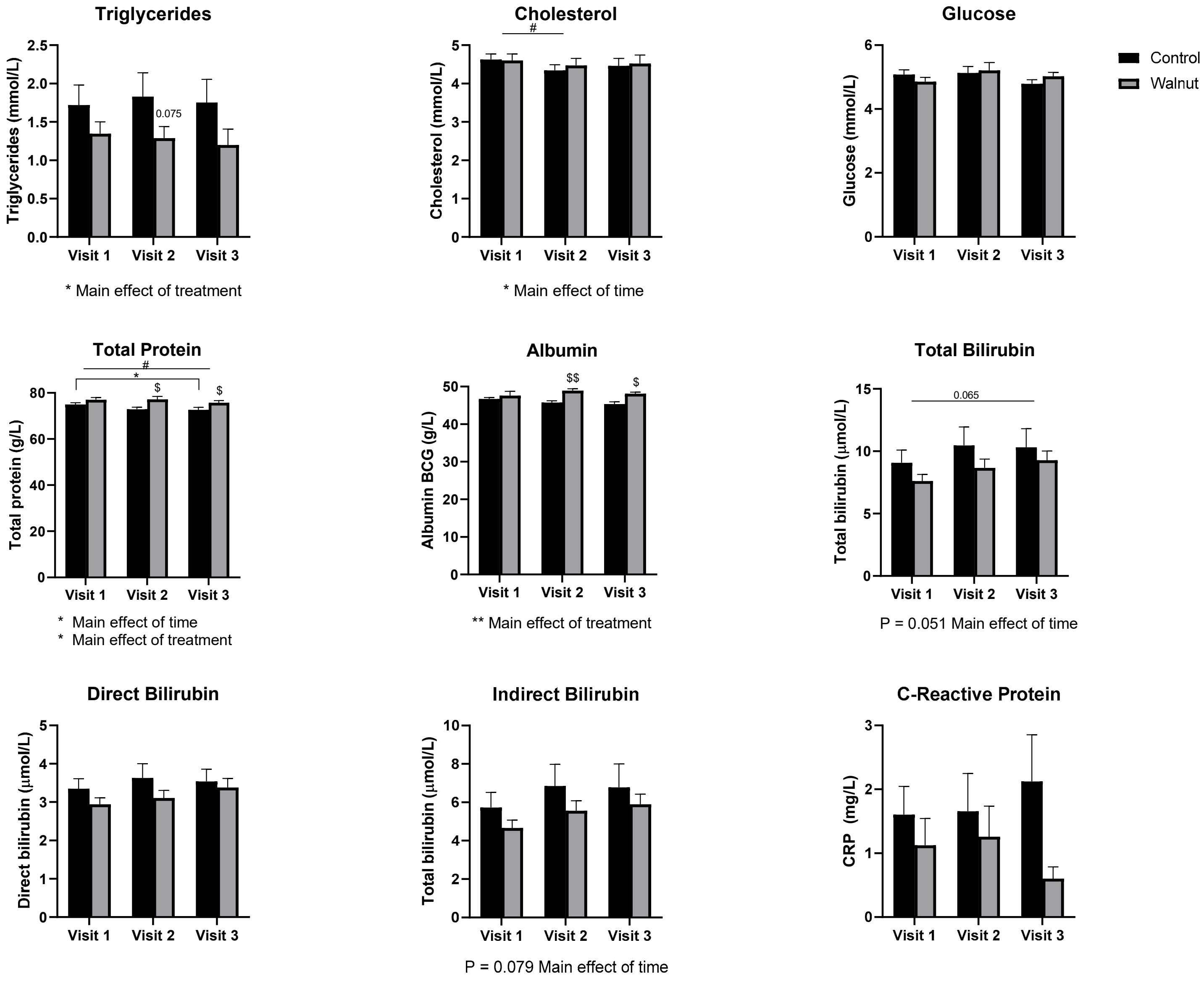

Figure S2. The effects of walnut consumption on biomarkers of general health in blood serum before, during and after the university examination period. Clinical Visit 1 occurred during the first half of the university semester, Visit 2 occurred during the university examination period, and Visit 3 occurred at the end of the university semester. Mixed effects analyses followed by Bonferroni multiple comparisons. * (Below graph) Denotes significant main effects, * = p < 0.05, ** = p < 0.01.* (On graph) Denotes a difference in control means compared to the immediately preceding visit unless otherwise indicated by lines, * = p < 0.05; $ Denotes a difference in means between the control and treatment (walnut) group at by visit, $ = p < 0.05, $$ = p < 0.01; p = 0.075 vs. control group at visit 2; # denotes a difference in main effect of time (visit), # = p < 0.05. Mixed effects analyses followed by Bonferroni multiple comparisons. CRP, C-reactive protein. Refer to Table 2 in main text for group sample numbers for each biomarker.

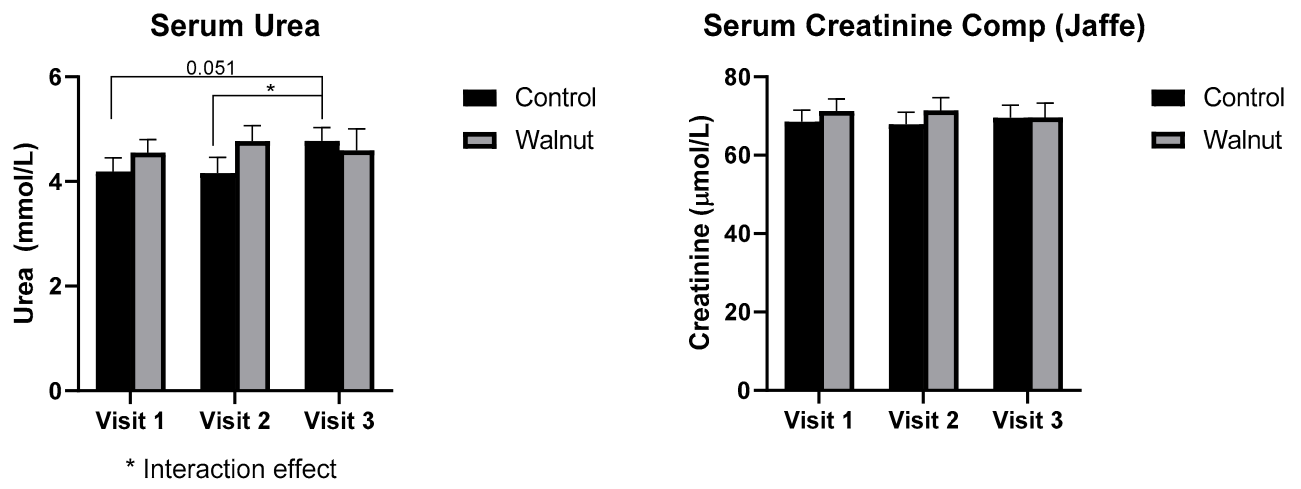

Figure S3. The effects of walnut consumption on biomarkers of general kidney function in blood serum before, during and after the university examination period. Clinical Visit 1 occurred during the first half of the university semester, Visit 2 occurred during the university examination period, and Visit 3 occurred at the end of the university semester. Mixed effects analyses followed by Bonferroni multiple comparisons. * (Below graph) Denotes significant main effects, * = $p < 0.05$.* (On graph) Denotes a difference in control means compared to the immediately preceding visit unless otherwise indicated by lines, * = $p < 0.05$. Creatinine composition evaluated using the Jaffe method. Urea – Visit 1: control n = 29, walnut n = 26; Urea – Visit 2: control n = 26, walnut n = 25; Urea – Visit 3: control n = 24, walnut n = 19. Creatinine Comp. – Visit 1: control n = 30, walnut n = 37; Creatinine Comp. – Visit 2: control n = 26, walnut n = 27; Creatinine Comp. – Visit 3: control n = 24, walnut n = 23. Small differences in sample size are due to technical issues with blood collection and withdrawals from subsequent visits. Analyses performed on samples from all 3 cohorts.

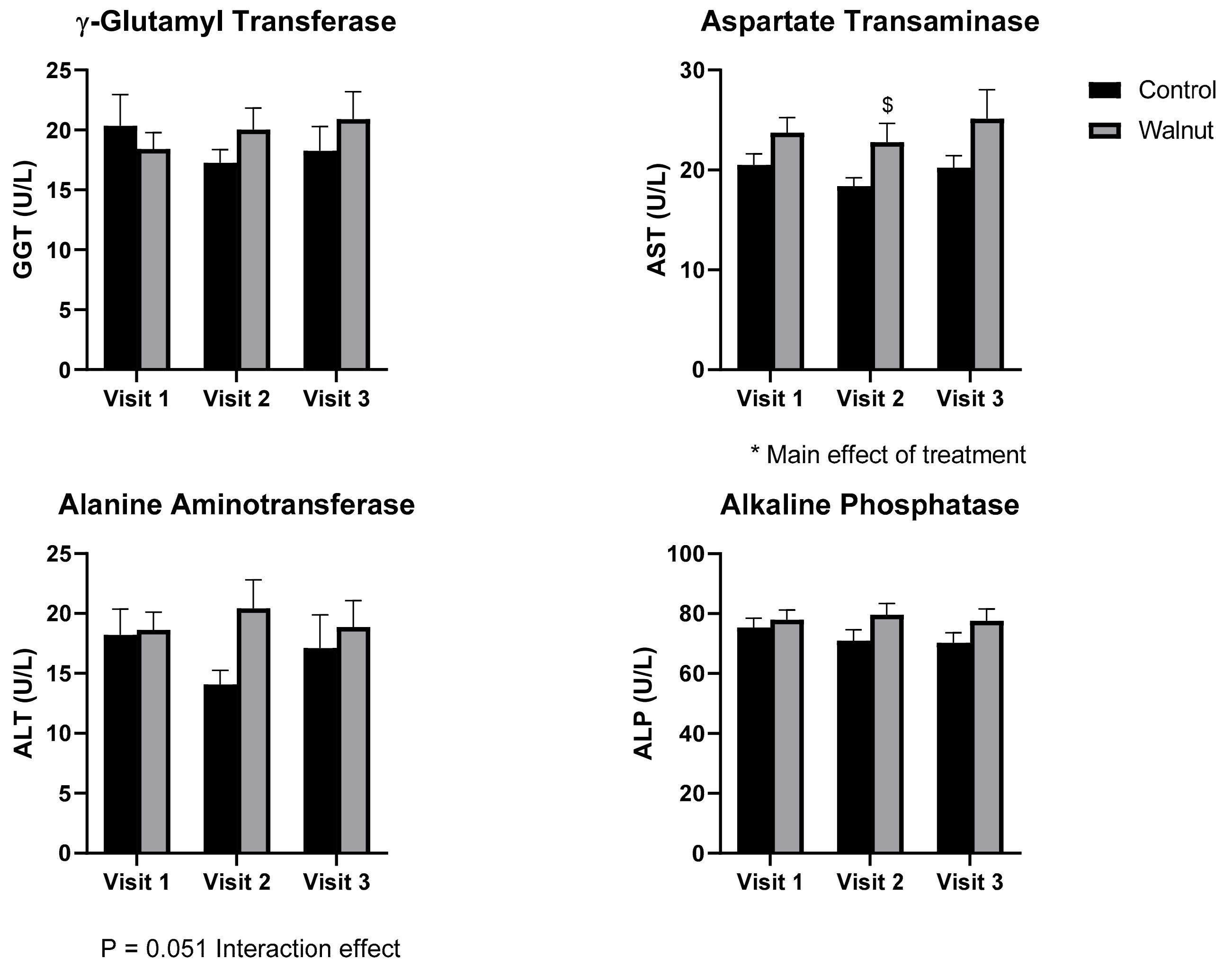

Figure S4. The effects of walnut consumption on biomarkers of general liver function in blood serum before, during and after the university examination period. Clinical Visit 1 occurred during the first half of the university semester, Visit 2 occurred during the university examination period, and Visit 3 occurred at the end of the university semester. Mixed effects analyses followed by Bonferroni multiple comparisons. * (Below graph) Denotes significant main effects, * = $p < 0.05$. $ Denotes a difference in means between the control and treatment (walnut) group at visit 2, $ = $p < 0.05$. GGT, γ-glutamyl transferase; AST, aspartate transaminase; ALT, alanine aminotransferase; ALP, alkaline phosphatase. GGT – Visit 1: control n = 30, walnut n = 37; GGT – Visit 2: control n = 26, walnut n = 27; GGT – Visit 3: control n = 24, walnut n = 22. AST. – Visit 1: control n = 30, walnut n = 37; AST – Visit 2: control n = 26, walnut n = 23; AST – Visit 3: control n = 24, walnut n = 21. ALT. – Visit 1: control n = 31, walnut n = 37; ALT – Visit 2: control n = 26, walnut n = 26; ALT – Visit 3: control n = 24, walnut n = 22. ALP. – Visit 1: control n = 30, walnut n = 37; ALP – Visit 2: control n = 25, walnut n = 27; ALP – Visit 3: control n = 24, walnut n = 23. Small differences in sample size are due to technical issues with blood collection and withdrawals from subsequent visits. Analyses performed on samples from all 3 cohorts.

Table S1. The effects of walnut consumption on total polyphenol content and antioxidant capacity of blood plasma before, during and after the university examination period. Clinical Visit 1 occurred during the first half of the university semester, Visit 2 occurred during the university examination period, and Visit 3 occurred at the end of the university semester. Mixed effects analyses followed by Bonferroni multiple comparisons comparing the control and walnut group at each clinical visit are shown. GAE, gallic acid equivalent; AA, ascorbic acid; TEq, Trolox equivalent. †.

	**Control diet**			**Walnut diet**			**P value (Control versus Walnut)**		
	**Visit 1**	**Visit 2**	**Visit 3**	**Visit 1**	**Visit 2**	**Visit 3**	**Visit 1**	**Visit 2**	**Visit 3**
Total polyphenol content (μg GAE/ml)	1608.941 ± 47.00 (n = 25)	1513.031 ± 45.39 (n = 24)	1706.030 ± 106.67 (n = 20)	1700.123 ± 77.27 (n = 24)	1662.863 ± 41.94 (n = 25)	1585.512 ± 62.99 (n = 18)	0.9592	0.0578	>0.9999
Ferric reducing ability of plasma (μg AA/ml)	1421.256 ± 15.84 (n = 26)	1357.246 ± 49.12 (n = 26)	1372.314 ± 28.37 (n = 21)	1437.826 ± 19.19 (n = 25)	1423.333 ± 20.24 (n = 25)	1431.871 ± 22.51 (n = 19)	>0.9999	0.6667	0.3256
Oxygen radical absorbance capacity (μmol TE/mg)	12394.230 ± 351.93 (n = 26)	12275.203 ± 462.36 (n = 26)	12696.725 ± 426.95 (n = 21)	12211.568 ± 393.09 (n = 25)	11765.261 ± 485.19 (n = 25)	485.190 ± 643.28 (n = 19)	>0.9999	>0.9999	0.9858

Small differences in sample size are due to technical issues with blood collection and withdrawals from subsequent visits. Analyses only performed on samples from cohort 1 and 2.

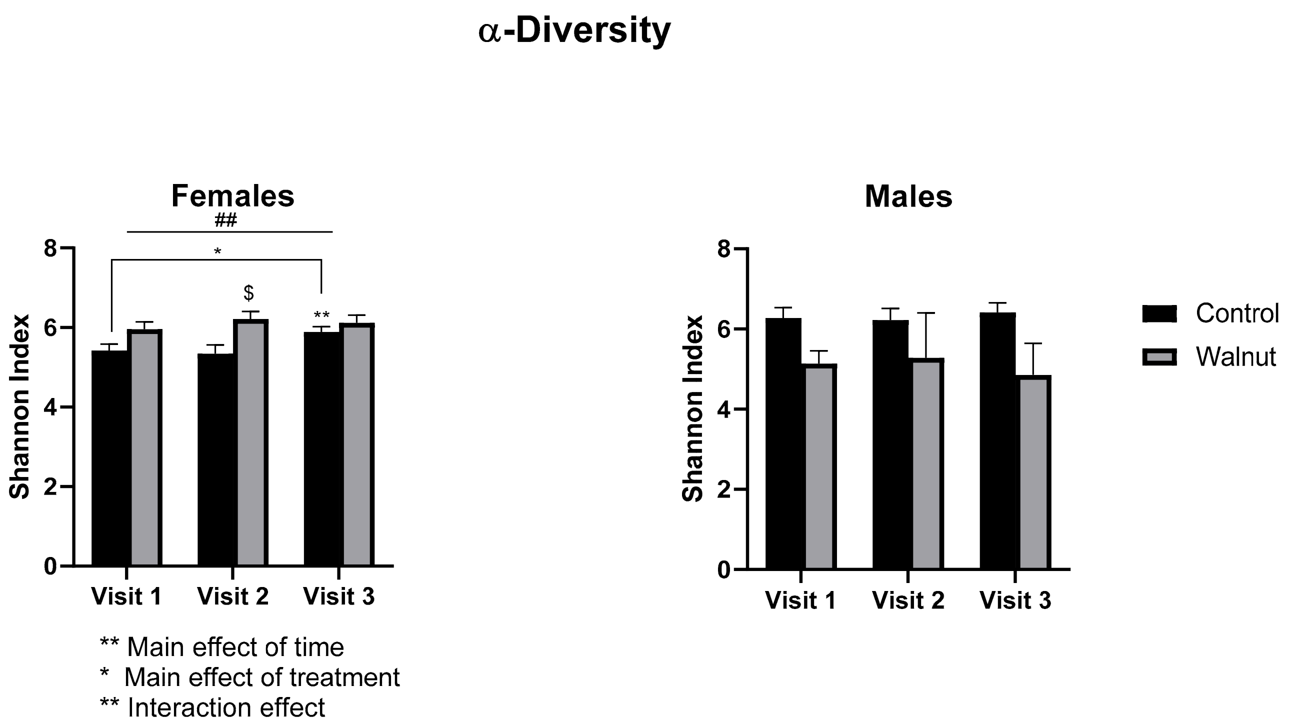

Figure S5. α-Diversity analysis of the gut microbiota in cohort 2 & 3 in females versus males before, during and after the university examination period. α-Diversity is represented by the Shannon diversity index. Clinical Visit 1 occurred during the first half of the university semester, Visit 2 occurred during the university examination period, and Visit 3 occurred at the end of the university semester. Mixed effects analyses followed by Bonferroni multiple comparisons. * (Below graph) Denotes significant main effects, * = $p < 0.05$, ** = $p < 0.01$. * (On graph) Denotes a difference in control means compared to the immediately preceding visit unless otherwise indicated by lines, * = $p < 0.05$, ** = $p < 0.01$. $ Denotes a difference in means between the control and treatment (walnut) group, $ = $p < 0.05$. # denotes a difference in main effect of time (visit), ## = $p < 0.01$. Females, Visit 1: control n = 14; walnut n = 11. Visit 2: control n = 11; walnut n = 10; Visit 3: control n = 11; walnut n = 9. Males, Visit 1: control n = 3; walnut n = 5. Visit 2: control n = 3; walnut n = 2; Visit 3: control n = 3; walnut n = 2. Results from cohort 2 and 3 are pooled.

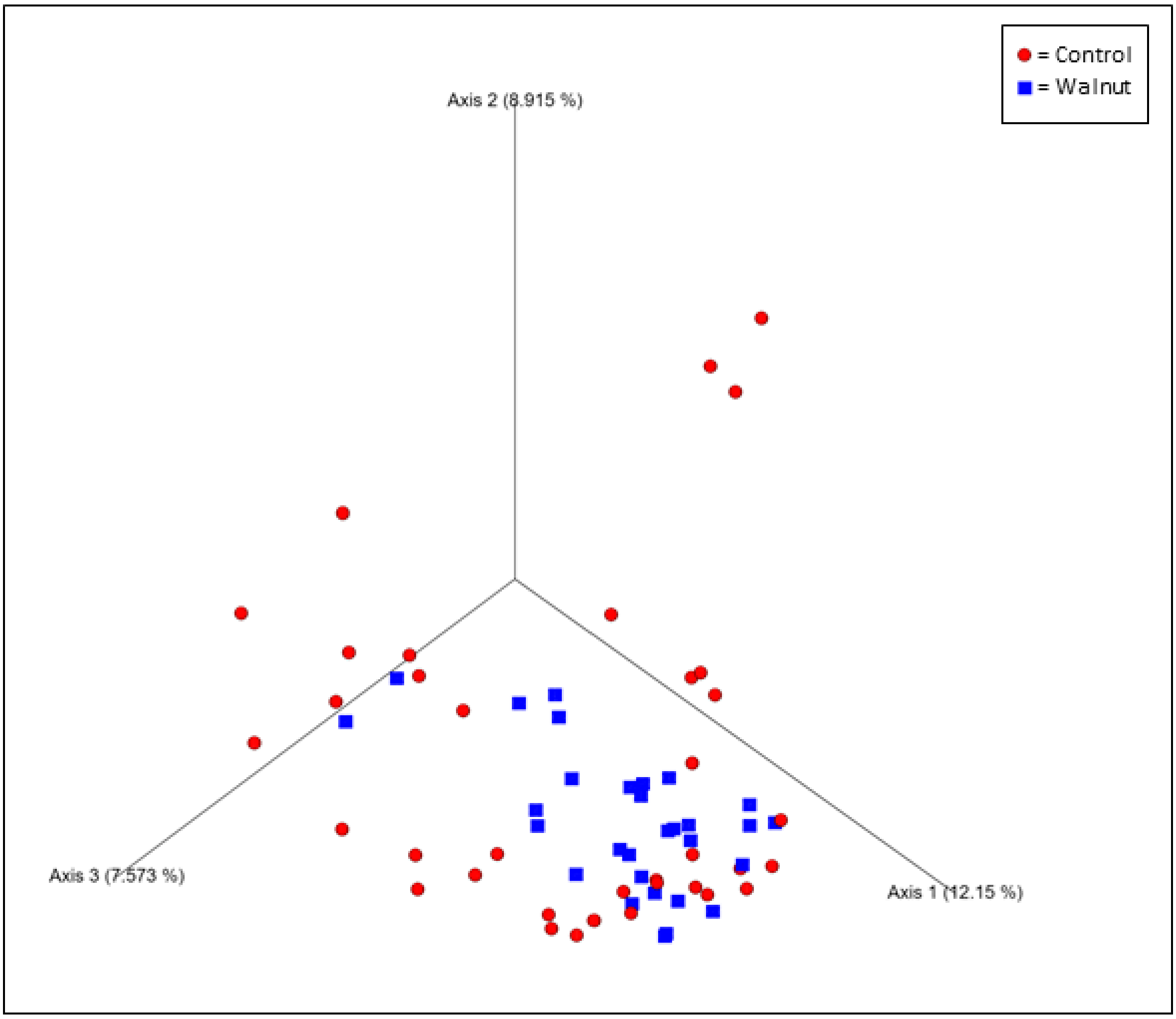

Figure S6. Principle coordinate analysis (PCoA) plot of Bray-Curtis dissimilarity in the gut microbiota in cohort 2 & 3 in females. Control, n = 36; Walnut, n = 30. Results from cohort 2 and 3 are pooled.

Table S2. β-Diversity analysis of the gut microbiota in cohort 2 & 3 in males before, during and after the university examination period. β - Diversity is represented by the Bray-Curtis dissimilarity index. Clinical Visit 1 occurred during the first half of the university semester, Visit 2 occurred during the university examination period, and Visit 3 occurred at the end of the university semester. Pairwise PERMANOVA comparisons are shown. * Denotes a difference in control versus walnut means per visit, * = $p < 0.05$. Visit 1: control n = 3; walnut n = 5. Visit 2: control n = 3; walnut n = 2; Visit 3: control n = 3; walnut n = 2. Results from cohort 2 and 3 are pooled.

Comparison 1	Comparison 2	Sample size	Permutations	pseudo-F	p-value	q-value
Control Visit 1	Control Visit 2	6	999	0.241876	0.79	1
Control Visit 1	Control Visit 3	6	999	0.23223	0.702	1
Control Visit 1	Walnut Visit 1	8	999	1.355055	0.042 *	0.36
Control Visit 1	Walnut Visit 2	5	999	0.846519	0.815	1
Control Visit 1	Walnut Visit 3	5	999	1.191944	0.304	0.8025
Control Visit 2	Control Visit 3	6	999	0.1401	1	1
Control Visit 2	Walnut Visit 1	8	999	1.436977	0.068	0.36
Control Visit 2	Walnut Visit 2	5	999	0.870726	0.808	1
Control Visit 2	Walnut Visit 3	5	999	1.268063	0.315	0.8025
Control Visit 3	Walnut Visit 1	8	999	1.384651	0.072	0.36
Control Visit 3	Walnut Visit 2	5	999	0.874581	0.799	1
Control Visit 3	Walnut Visit 3	5	999	1.213358	0.321	0.8025
Walnut Visit 1	Walnut Visit 2	7	999	0.576801	0.949	1
Walnut Visit 1	Walnut Visit 3	7	999	0.534501	1	1
Walnut Visit 2	Walnut Visit 3	4	999	0.279272	1	1

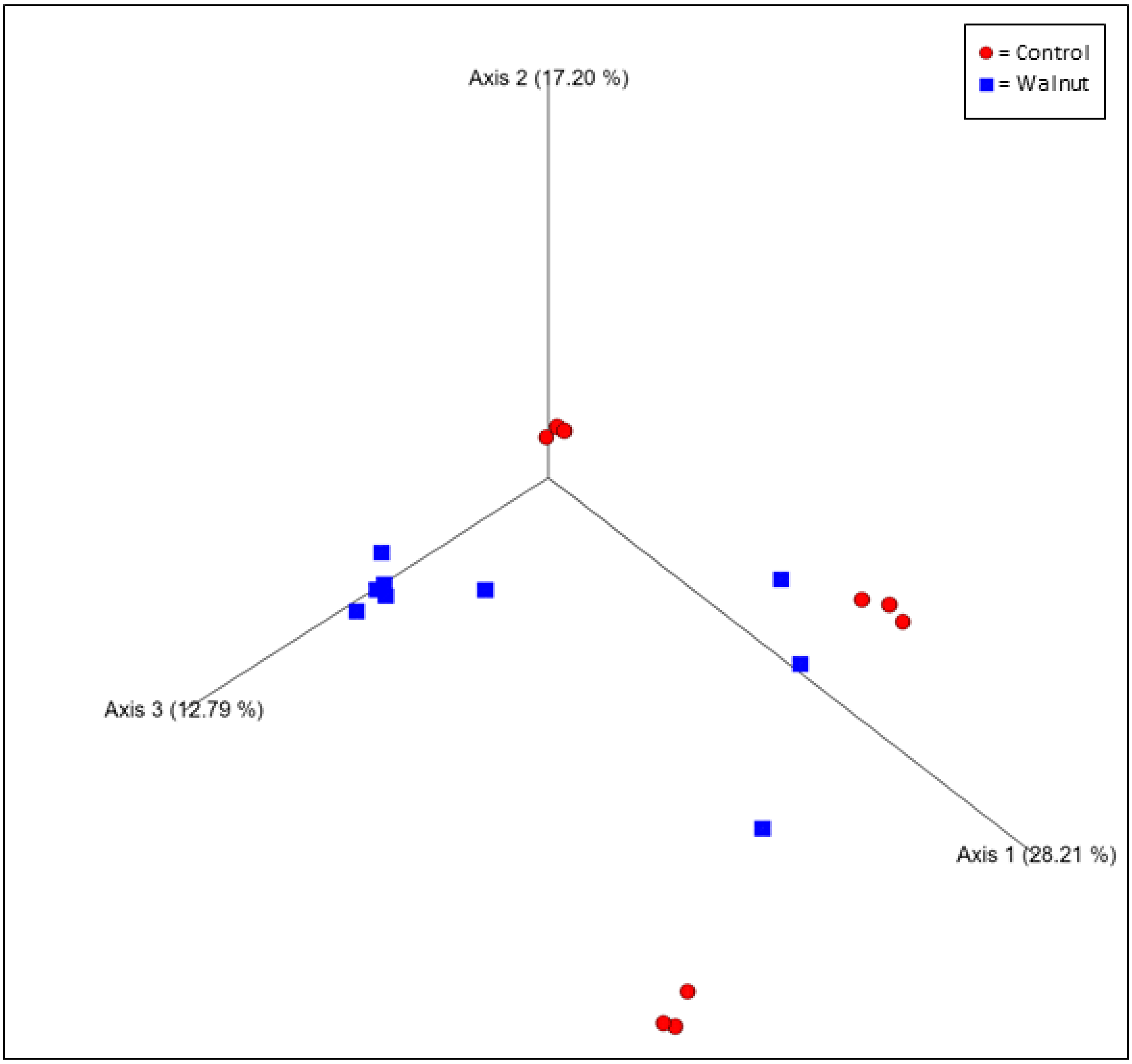

Figure S7. Principle coordinate analysis (PCoA) plot of Bray-Curtis dissimilarity in the gut microbiota in cohort 2 & 3 in males. Control, n = 9; Walnut, n = 9. Results from cohort 2 and 3 are pooled.

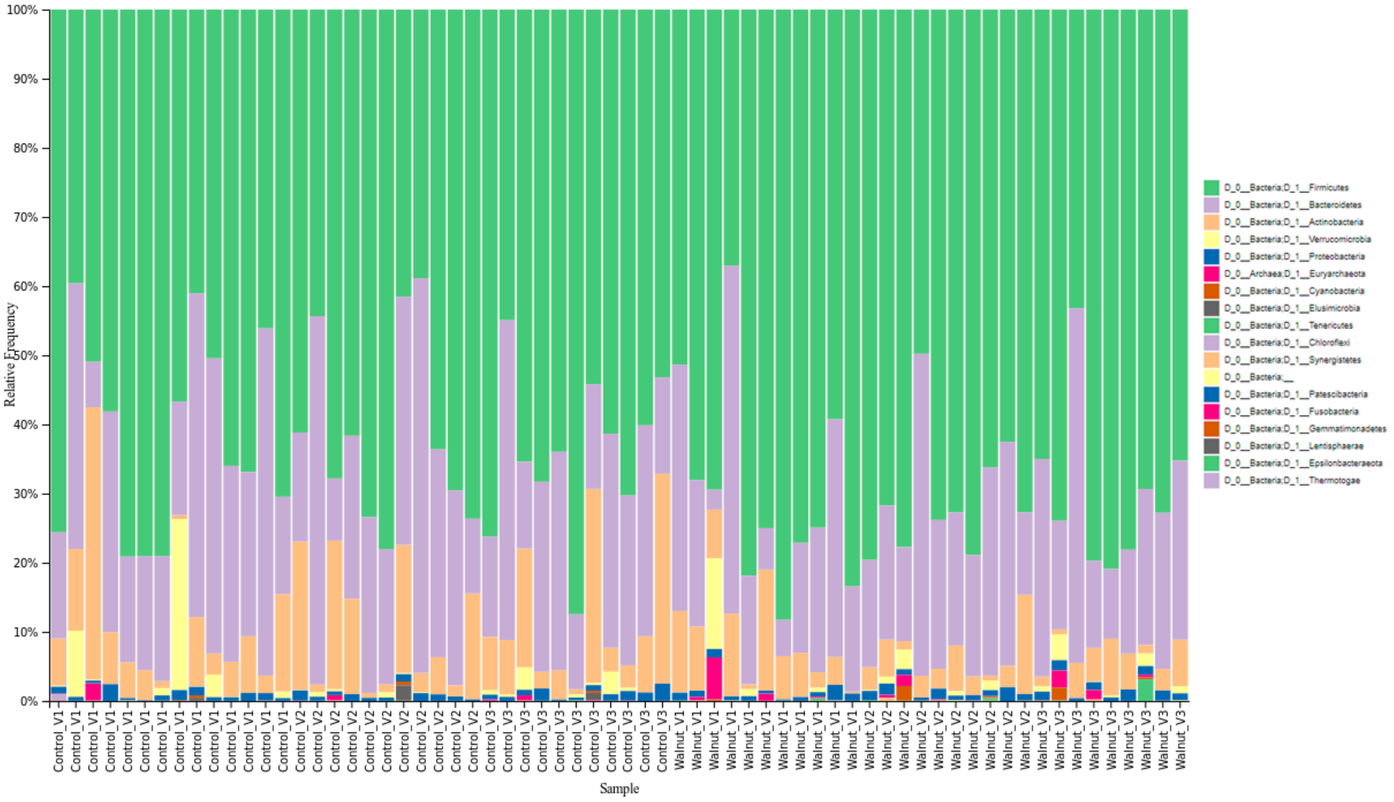

Figure S8. Bar plot showing the relative taxonomic frequency of the gut microbiota at the phylum level in females before, during and after the university examination period. Clinical Visit 1 (V1) occurred during the first half of the university semester, Visit 2 (V2) occurred during the university examination period, and Visit 3 (V3) occurred at the end of the university semester. Visit 1: control n = 14; walnut n = 11. Visit 2: control n = 11; walnut n = 10; Visit 3: control n = 11; walnut n = 9. Results from cohort 2 and 3 are pooled.

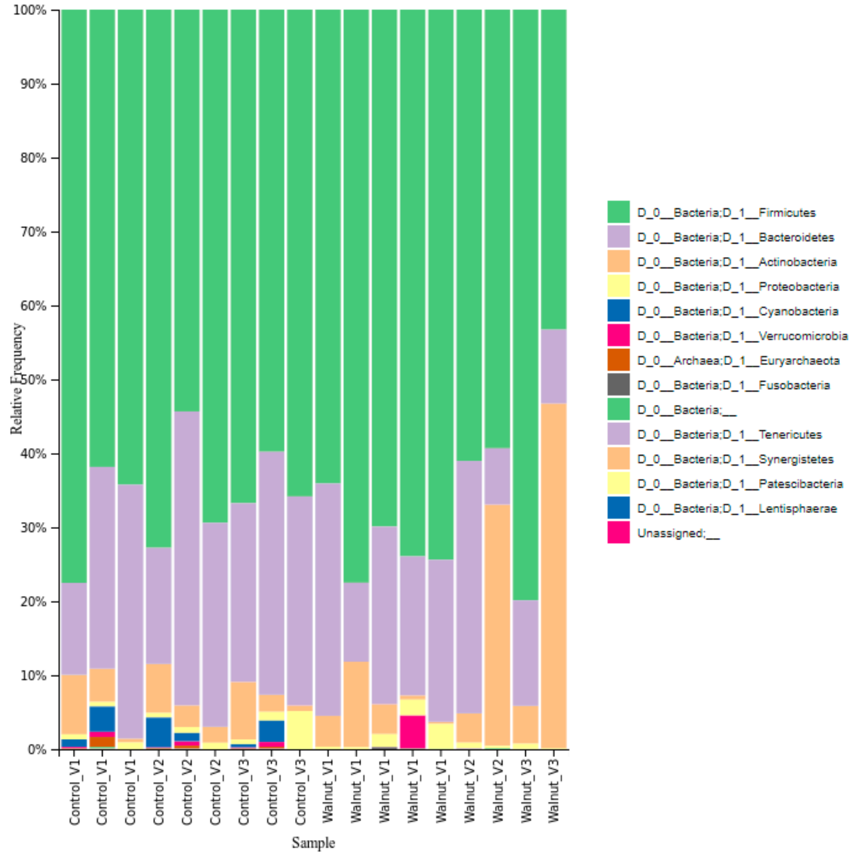

Figure S9. Bar plot showing the relative taxonomic frequency of the gut microbiota at the phylum level in males before, during and after the university examination period. Clinical Visit 1 (V1) occurred during the first half of the university semester, Visit 2 (V2) occurred during the university examination period, and Visit 3 (V3) occurred at the end of the university semester. Visit 1: control n = 3; walnut n = 5. Visit 2: control n = 3; walnut n = 2; Visit 3: control n = 3; walnut n = 2. Results from cohort 2 and 3 are pooled.

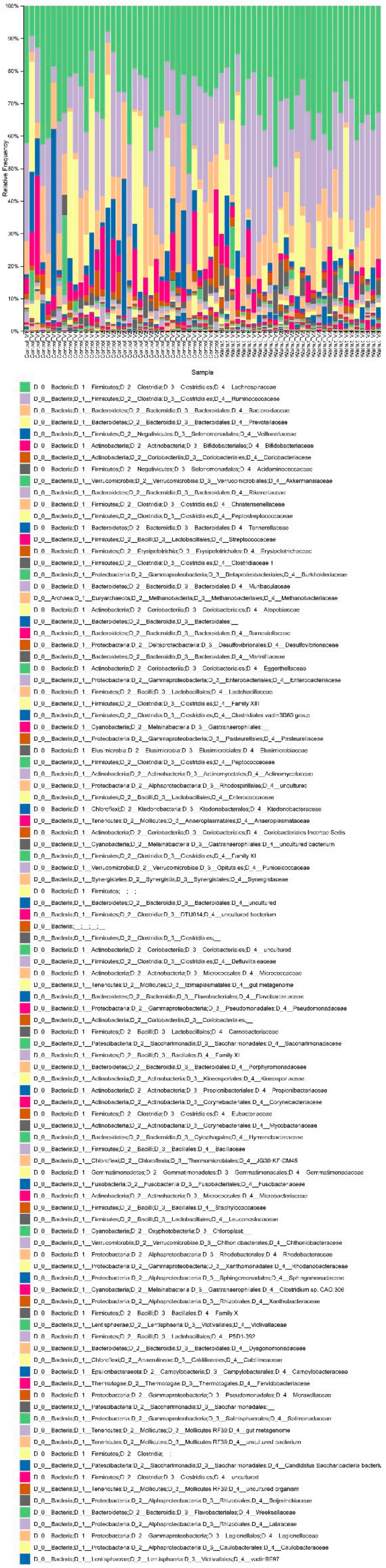

100%
90%
80%
70%
60%
50%
40%
30%
20%
10%
0%
Relative Frequency
Sample
D_0__Bacteria;D_1__Firmicutes;D_2__Clostridia;D_3__Clostridiales;D_4__Lachnospiraceae
D_0__Bacteria;D_1__Firmicutes;D_2__Clostridia;D_3__Clostridiales;D_4__Ruminococcaceae
D_0__Bacteria;D_1__Bacteroidetes;D_2__Bacteroidia;D_3__Bacteroidales;D_4__Bacteroidaceae
D_0__Bacteria;D_1__Bacteroidetes;D_2__Bacteroidia;D_3__Bacteroidales;D_4__Prevotellaceae
D_0__Bacteria;D_1__Firmicutes;D_2__Negativicutes;D_3__Selenomonadales;D_4__Veillonellaceae
D_0__Bacteria;D_1__Actinobacteria;D_2__Actinobacteria;D_3__Bifidobacteriales;D_4__Bifidobacteriaceae
D_0__Bacteria;D_1__Actinobacteria;D_2__Coriobacteriia;D_3__Coriobacteriales;D_4__Coriobacteriaceae
D_0__Bacteria;D_1__Firmicutes;D_2__Negativicutes;D_3__Selenomonadales;D_4__Acidaminococcaceae
D_0__Bacteria;D_1__Verrucomicrobia;D_2__Verrucomicrobiae;D_3__Verrucomicrobiales;D_4__Akkermansiaceae
D_0__Bacteria;D_1__Bacteroidetes;D_2__Bacteroidia;D_3__Bacteroidales;D_4__Rikenellaceae
D_0__Bacteria;D_1__Firmicutes;D_2__Clostridia;D_3__Clostridiales;D_4__Christensenellaceae
D_0__Bacteria;D_1__Firmicutes;D_2__Clostridia;D_3__Clostridiales;D_4__Peptostreptococcaceae
D_0__Bacteria;D_1__Bacteroidetes;D_2__Bacteroidia;D_3__Bacteroidales;D_4__Tannerellaceae
D_0__Bacteria;D_1__Firmicutes;D_2__Bacilli;D_3__Lactobacillales;D_4__Streptococcaceae
D_0__Bacteria;D_1__Firmicutes;D_2__Erysipelotrichia;D_3__Erysipelotrichales;D_4__Erysipelotrichaceae
D_0__Bacteria;D_1__Firmicutes;D_2__Clostridia;D_3__Clostridiales;D_4__Clostridiaceae 1
D_0__Bacteria;D_1__Proteobacteria;D_2__Gammaproteobacteria;D_3__Betaproteobacteriales;D_4__Burkholderiaceae
D_0__Bacteria;D_1__Bacteroidetes;D_2__Bacteroidia;D_3__Bacteroidales;D_4__Muribaculaceae
D_0__Archaea;D_1__Euryarchaeota;D_2__Methanobacteria;D_3__Methanobacteriales;D_4__Methanobacteriaceae
D_0__Bacteria;D_1__Actinobacteria;D_2__Coriobacteriia;D_3__Coriobacteriales;D_4__Atopobiaceae
D_0__Bacteria;D_1__Bacteroidetes;D_2__Bacteroidia;D_3__Bacteroidales;__
D_0__Bacteria;D_1__Bacteroidetes;D_2__Bacteroidia;D_3__Bacteroidales;D_4__Barnesiellaceae
D_0__Bacteria;D_1__Proteobacteria;D_2__Deltaproteobacteria;D_3__Desulfovibrionales;D_4__Desulfovibrionaceae
D_0__Bacteria;D_1__Bacteroidetes;D_2__Bacteroidia;D_3__Bacteroidales;D_4__Marinifilaceae
D_0__Bacteria;D_1__Actinobacteria;D_2__Coriobacteriia;D_3__Coriobacteriales;D_4__Eggerthellaceae
D_0__Bacteria;D_1__Proteobacteria;D_2__Gammaproteobacteria;D_3__Enterobacteriales;D_4__Enterobacteriaceae
D_0__Bacteria;D_1__Firmicutes;D_2__Bacilli;D_3__Lactobacillales;D_4__Lactobacillaceae
D_0__Bacteria;D_1__Firmicutes;D_2__Clostridia;D_3__Clostridiales;D_4__Family XIII
D_0__Bacteria;D_1__Firmicutes;D_2__Clostridia;D_3__Clostridiales;D_4__Clostridiales vadinBB60 group
D_0__Bacteria;D_1__Cyanobacteria;D_2__Melainabacteria;D_3__Gastranaerophilales;__
D_0__Bacteria;D_1__Proteobacteria;D_2__Gammaproteobacteria;D_3__Pasteurellales;D_4__Pasteurellaceae
D_0__Bacteria;D_1__Elusimicrobia;D_2__Elusimicrobia;D_3__Elusimicrobiales;D_4__Elusimicrobiaceae
D_0__Bacteria;D_1__Firmicutes;D_2__Clostridia;D_3__Clostridiales;D_4__Peptococcaceae
D_0__Bacteria;D_1__Actinobacteria;D_2__Actinobacteria;D_3__Actinomycetales;D_4__Actinomycetaceae
D_0__Bacteria;D_1__Proteobacteria;D_2__Alphaproteobacteria;D_3__Rhodospirillales;D_4__uncultured
D_0__Bacteria;D_1__Firmicutes;D_2__Bacilli;D_3__Lactobacillales;D_4__Enterococcaceae
D_0__Bacteria;D_1__Chloroflexi;D_2__Ktedonobacteria;D_3__Ktedonobacterales;D_4__Ktedonobacteraceae
D_0__Bacteria;D_1__Tenericutes;D_2__Mollicutes;D_3__Anaeroplasmatales;D_4__Anaeroplasmataceae
D_0__Bacteria;D_1__Actinobacteria;D_2__Coriobacteriia;D_3__Coriobacteriales;D_4__Coriobacteriales Incertae Sedis
D_0__Bacteria;D_1__Cyanobacteria;D_2__Melainabacteria;D_3__Gastranaerophilales;D_4__uncultured bacterium
D_0__Bacteria;D_1__Firmicutes;D_2__Clostridia;D_3__Clostridiales;D_4__Family XI
D_0__Bacteria;D_1__Verrucomicrobia;D_2__Verrucomicrobiae;D_3__Opitutales;D_4__Puniceicoccaceae
D_0__Bacteria;D_1__Synergistetes;D_2__Synergistia;D_3__Synergistales;D_4__Synergistaceae
D_0__Bacteria;D_1__Firmicutes;__;__;__
D_0__Bacteria;D_1__Bacteroidetes;D_2__Bacteroidia;D_3__Bacteroidales;D_4__uncultured
D_0__Bacteria;D_1__Firmicutes;D_2__Clostridia;D_3__DTU014;D_4__uncultured bacterium
D_0__Bacteria;__;__;__;__
D_0__Bacteria;D_1__Firmicutes;D_2__Clostridia;D_3__Clostridiales;__
D_0__Bacteria;D_1__Actinobacteria;D_2__Coriobacteriia;D_3__Coriobacteriales;D_4__uncultured
D_0__Bacteria;D_1__Firmicutes;D_2__Clostridia;D_3__Clostridiales;D_4__Defluviitaleaceae
D_0__Bacteria;D_1__Actinobacteria;D_2__Actinobacteria;D_3__Micrococcales;D_4__Micrococcaceae
D_0__Bacteria;D_1__Tenericutes;D_2__Mollicutes;D_3__Izimaplasmatales;D_4__gut metagenome
D_0__Bacteria;D_1__Bacteroidetes;D_2__Bacteroidia;D_3__Flavobacteriales;D_4__Flavobacteriaceae
D_0__Bacteria;D_1__Proteobacteria;D_2__Gammaproteobacteria;D_3__Pseudomonadales;D_4__Pseudomonadaceae
D_0__Bacteria;D_1__Actinobacteria;D_2__Coriobacteriia;D_3__Coriobacteriales;__
D_0__Bacteria;D_1__Firmicutes;D_2__Bacilli;D_3__Lactobacillales;D_4__Carnobacteriaceae
D_0__Bacteria;D_1__Patescibacteria;D_2__Saccharimonadia;D_3__Saccharimonadales;D_4__Saccharimonadaceae
D_0__Bacteria;D_1__Firmicutes;D_2__Bacilli;D_3__Bacillales;D_4__Family XI
D_0__Bacteria;D_1__Bacteroidetes;D_2__Bacteroidia;D_3__Bacteroidales;D_4__Porphyromonadaceae
D_0__Bacteria;D_1__Actinobacteria;D_2__Actinobacteria;D_3__Kineosporiales;D_4__Kineosporiaceae
D_0__Bacteria;D_1__Actinobacteria;D_2__Actinobacteria;D_3__Propionibacteriales;D_4__Propionibacteriaceae
D_0__Bacteria;D_1__Actinobacteria;D_2__Actinobacteria;D_3__Corynebacteriales;D_4__Corynebacteriaceae
D_0__Bacteria;D_1__Firmicutes;D_2__Clostridia;D_3__Clostridiales;D_4__Eubacteriaceae
D_0__Bacteria;D_1__Actinobacteria;D_2__Actinobacteria;D_3__Corynebacteriales;D_4__Mycobacteriaceae
D_0__Bacteria;D_1__Bacteroidetes;D_2__Bacteroidia;D_3__Cytophagales;D_4__Hymenobacteraceae
D_0__Bacteria;D_1__Firmicutes;D_2__Bacilli;D_3__Bacillales;D_4__Bacillaceae
D_0__Bacteria;D_1__Chloroflexi;D_2__Chloroflexia;D_3__Thermomicrobiales;D_4__JG30-KF-CM45
D_0__Bacteria;D_1__Gemmatimonadetes;D_2__Gemmatimonadetes;D_3__Gemmatimonadales;D_4__Gemmatimonadaceae
D_0__Bacteria;D_1__Fusobacteria;D_2__Fusobacteriia;D_3__Fusobacteriales;D_4__Fusobacteriaceae
D_0__Bacteria;D_1__Actinobacteria;D_2__Actinobacteria;D_3__Micrococcales;D_4__Microbacteriaceae
D_0__Bacteria;D_1__Firmicutes;D_2__Bacilli;D_3__Bacillales;D_4__Staphylococcaceae
D_0__Bacteria;D_1__Firmicutes;D_2__Bacilli;D_3__Lactobacillales;D_4__Leuconostocaceae
D_0__Bacteria;D_1__Cyanobacteria;D_2__Oxyphotobacteria;D_3__Chloroplast;__
D_0__Bacteria;D_1__Verrucomicrobia;D_2__Verrucomicrobiae;D_3__Chthoniobacterales;D_4__Chthoniobacteraceae
D_0__Bacteria;D_1__Proteobacteria;D_2__Alphaproteobacteria;D_3__Rhodobacterales;D_4__Rhodobacteraceae
D_0__Bacteria;D_1__Proteobacteria;D_2__Gammaproteobacteria;D_3__Xanthomonadales;D_4__Rhodanobacteraceae
D_0__Bacteria;D_1__Proteobacteria;D_2__Alphaproteobacteria;D_3__Sphingomonadales;D_4__Sphingomonadaceae
D_0__Bacteria;D_1__Cyanobacteria;D_2__Melainabacteria;D_3__Gastranaerophilales;D_4__Clostridium sp. CAG:306
D_0__Bacteria;D_1__Proteobacteria;D_2__Alphaproteobacteria;D_3__Rhizobiales;D_4__Xanthobacteraceae
D_0__Bacteria;D_1__Firmicutes;D_2__Bacilli;D_3__Bacillales;D_4__Family X
D_0__Bacteria;D_1__Lentisphaerae;D_2__Lentisphaeria;D_3__Victivallales;D_4__Victivallaceae
D_0__Bacteria;D_1__Firmicutes;D_2__Bacilli;D_3__Lactobacillales;D_4__P5D1-392
D_0__Bacteria;D_1__Bacteroidetes;D_2__Bacteroidia;D_3__Bacteroidales;D_4__Dysgonomonadaceae
D_0__Bacteria;D_1__Chloroflexi;D_2__Anaerolineae;D_3__Caldilineales;D_4__Caldilineaceae
D_0__Bacteria;D_1__Epsilonbacteraeota;D_2__Campylobacteria;D_3__Campylobacterales;D_4__Campylobacteraceae
D_0__Bacteria;D_1__Thermotogae;D_2__Thermotogae;D_3__Thermotogales;D_4__Fervidobacteriaceae
D_0__Bacteria;D_1__Proteobacteria;D_2__Gammaproteobacteria;D_3__Pseudomonadales;D_4__Moraxellaceae
D_0__Bacteria;D_1__Patescibacteria;D_2__Saccharimonadia;D_3__Saccharimonadales;__
D_0__Bacteria;D_1__Proteobacteria;D_2__Gammaproteobacteria;D_3__Salinisphaerales;D_4__Solimonadaceae
D_0__Bacteria;D_1__Tenericutes;D_2__Mollicutes;D_3__Mollicutes RF39;D_4__gut metagenome
D_0__Bacteria;D_1__Tenericutes;D_2__Mollicutes;D_3__Mollicutes RF39;D_4__uncultured bacterium
D_0__Bacteria;D_1__Firmicutes;D_2__Clostridia;__;__
D_0__Bacteria;D_1__Patescibacteria;D_2__Saccharimonadia;D_3__Saccharimonadales;D_4__Candidatus Saccharibacteria bacteriu
D_0__Bacteria;D_1__Firmicutes;D_2__Clostridia;D_3__Clostridiales;D_4__uncultured
D_0__Bacteria;D_1__Tenericutes;D_2__Mollicutes;D_3__Mollicutes RF39;D_4__uncultured organism
D_0__Bacteria;D_1__Proteobacteria;D_2__Alphaproteobacteria;D_3__Rhizobiales;D_4__Beijerinckiaceae
D_0__Bacteria;D_1__Bacteroidetes;D_2__Bacteroidia;D_3__Flavobacteriales;D_4__Weeksellaceae
D_0__Bacteria;D_1__Proteobacteria;D_2__Alphaproteobacteria;D_3__Rhizobiales;D_4__Labraceae
D_0__Bacteria;D_1__Proteobacteria;D_2__Gammaproteobacteria;D_3__Legionellales;D_4__Legionellaceae
D_0__Bacteria;D_1__Proteobacteria;D_2__Alphaproteobacteria;D_3__Caulobacterales;D_4__Caulobacteraceae
D_0__Bacteria;D_1__Lentisphaerae;D_2__Lentisphaeria;D_3__Victivallales;D_4__vadinBE97

Figure S10. Bar plot showing the relative taxonomic frequency of the gut microbiota at the family level in females before, during and after the university examination period. Clinical Visit 1 (V1) occurred during the first half of the university semester, Visit 2 (V2) occurred during the university examination period, and Visit 3 (V3) occurred at the end of the university semester. Visit 1: control n = 14; walnut n = 11. Visit 2: control n = 11; walnut n = 10; Visit 3: control n = 11; walnut n = 9. Results from cohort 2 and 3 are pooled.

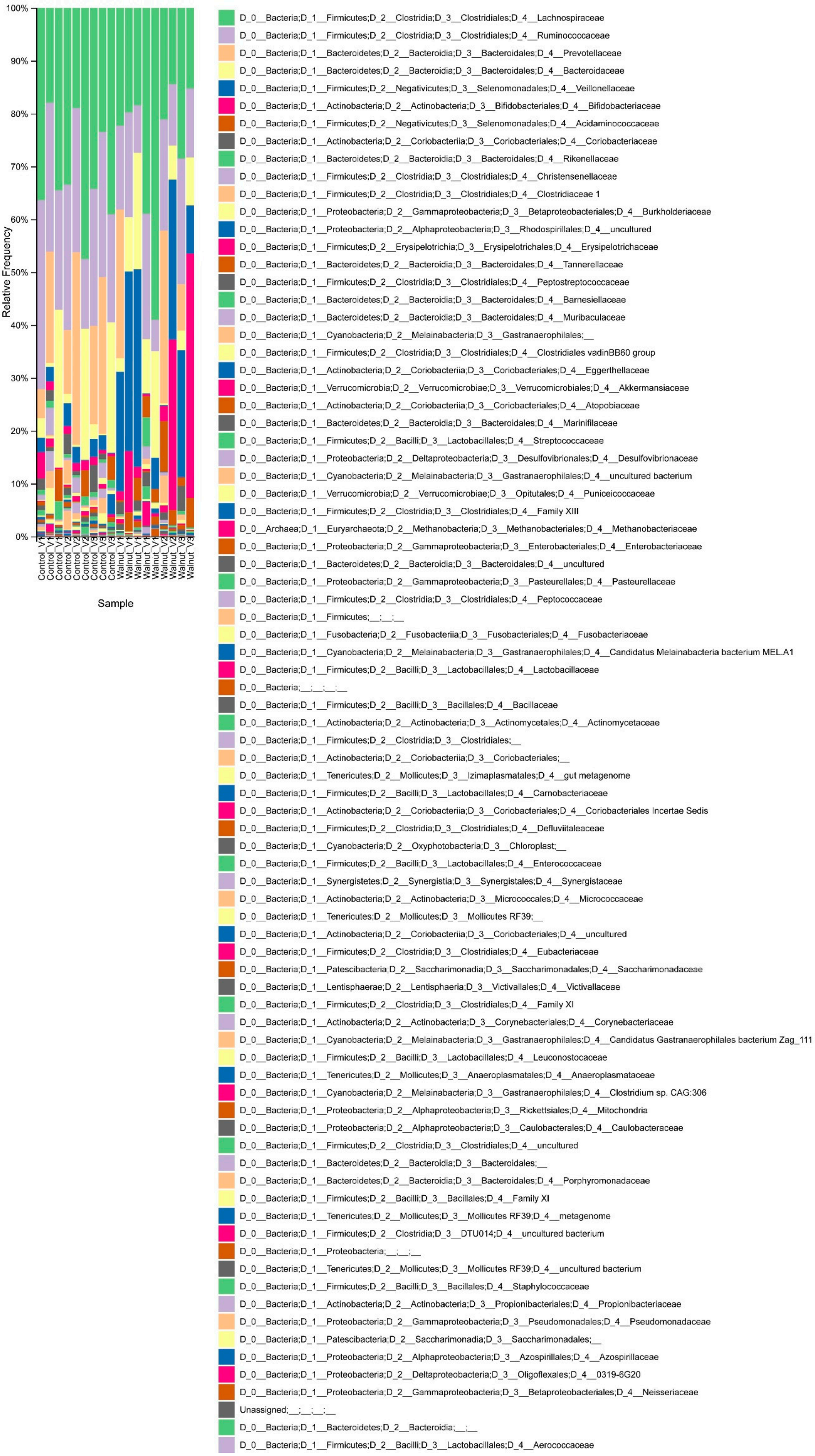

100%
90%
80%
70%
60%
50%
40%
30%
20%
10%
0%
Relative Frequency
Sample
D_0__Bacteria;D_1__Firmicutes;D_2__Clostridia;D_3__Clostridiales;D_4__Lachnospiraceae
D_0__Bacteria;D_1__Firmicutes;D_2__Clostridia;D_3__Clostridiales;D_4__Ruminococcaceae
D_0__Bacteria;D_1__Bacteroidetes;D_2__Bacteroidia;D_3__Bacteroidales;D_4__Prevotellaceae
D_0__Bacteria;D_1__Bacteroidetes;D_2__Bacteroidia;D_3__Bacteroidales;D_4__Bacteroidaceae
D_0__Bacteria;D_1__Firmicutes;D_2__Negativicutes;D_3__Selenomonadales;D_4__Veillonellaceae
D_0__Bacteria;D_1__Actinobacteria;D_2__Actinobacteria;D_3__Bifidobacteriales;D_4__Bifidobacteriaceae
D_0__Bacteria;D_1__Firmicutes;D_2__Negativicutes;D_3__Selenomonadales;D_4__Acidaminococcaceae
D_0__Bacteria;D_1__Actinobacteria;D_2__Coriobacteriia;D_3__Coriobacteriales;D_4__Coriobacteriaceae
D_0__Bacteria;D_1__Bacteroidetes;D_2__Bacteroidia;D_3__Bacteroidales;D_4__Rikenellaceae
D_0__Bacteria;D_1__Firmicutes;D_2__Clostridia;D_3__Clostridiales;D_4__Christensenellaceae
D_0__Bacteria;D_1__Firmicutes;D_2__Clostridia;D_3__Clostridiales;D_4__Clostridiaceae 1
D_0__Bacteria;D_1__Proteobacteria;D_2__Gammaproteobacteria;D_3__Betaproteobacteriales;D_4__Burkholderiaceae
D_0__Bacteria;D_1__Proteobacteria;D_2__Alphaproteobacteria;D_3__Rhodospirillales;D_4__uncultured
D_0__Bacteria;D_1__Firmicutes;D_2__Erysipelotrichia;D_3__Erysipelotrichales;D_4__Erysipelotrichaceae
D_0__Bacteria;D_1__Bacteroidetes;D_2__Bacteroidia;D_3__Bacteroidales;D_4__Tannerellaceae
D_0__Bacteria;D_1__Firmicutes;D_2__Clostridia;D_3__Clostridiales;D_4__Peptostreptococcaceae
D_0__Bacteria;D_1__Bacteroidetes;D_2__Bacteroidia;D_3__Bacteroidales;D_4__Barnesiellaceae
D_0__Bacteria;D_1__Bacteroidetes;D_2__Bacteroidia;D_3__Bacteroidales;D_4__Muribaculaceae
D_0__Bacteria;D_1__Cyanobacteria;D_2__Melainabacteria;D_3__Gastranaerophilales;__
D_0__Bacteria;D_1__Firmicutes;D_2__Clostridia;D_3__Clostridiales;D_4__Clostridiales vadinBB60 group
D_0__Bacteria;D_1__Actinobacteria;D_2__Coriobacteriia;D_3__Coriobacteriales;D_4__Eggerthellaceae
D_0__Bacteria;D_1__Verrucomicrobia;D_2__Verrucomicrobiae;D_3__Verrucomicrobiales;D_4__Akkermansiaceae
D_0__Bacteria;D_1__Actinobacteria;D_2__Coriobacteriia;D_3__Coriobacteriales;D_4__Atopobiaceae
D_0__Bacteria;D_1__Bacteroidetes;D_2__Bacteroidia;D_3__Bacteroidales;D_4__Marinifilaceae
D_0__Bacteria;D_1__Firmicutes;D_2__Bacilli;D_3__Lactobacillales;D_4__Streptococcaceae
D_0__Bacteria;D_1__Proteobacteria;D_2__Deltaproteobacteria;D_3__Desulfovibrionales;D_4__Desulfovibrionaceae
D_0__Bacteria;D_1__Cyanobacteria;D_2__Melainabacteria;D_3__Gastranaerophilales;D_4__uncultured bacterium
D_0__Bacteria;D_1__Verrucomicrobia;D_2__Verrucomicrobiae;D_3__Opitutales;D_4__Puniceicoccaceae
D_0__Bacteria;D_1__Firmicutes;D_2__Clostridia;D_3__Clostridiales;D_4__Family XIII
D_0__Archaea;D_1__Euryarchaeota;D_2__Methanobacteria;D_3__Methanobacteriales;D_4__Methanobacteriaceae
D_0__Bacteria;D_1__Proteobacteria;D_2__Gammaproteobacteria;D_3__Enterobacteriales;D_4__Enterobacteriaceae
D_0__Bacteria;D_1__Bacteroidetes;D_2__Bacteroidia;D_3__Bacteroidales;D_4__uncultured
D_0__Bacteria;D_1__Proteobacteria;D_2__Gammaproteobacteria;D_3__Pasteurellales;D_4__Pasteurellaceae
D_0__Bacteria;D_1__Firmicutes;D_2__Clostridia;D_3__Clostridiales;D_4__Peptococcaceae
D_0__Bacteria;D_1__Firmicutes;__;__;__
D_0__Bacteria;D_1__Fusobacteria;D_2__Fusobacteriia;D_3__Fusobacteriales;D_4__Fusobacteriaceae
D_0__Bacteria;D_1__Cyanobacteria;D_2__Melainabacteria;D_3__Gastranaerophilales;D_4__Candidatus Melainabacteria bacterium MEL.A1
D_0__Bacteria;D_1__Firmicutes;D_2__Bacilli;D_3__Lactobacillales;D_4__Lactobacillaceae
D_0__Bacteria;__;__;__;__
D_0__Bacteria;D_1__Firmicutes;D_2__Bacilli;D_3__Bacillales;D_4__Bacillaceae
D_0__Bacteria;D_1__Actinobacteria;D_2__Actinobacteria;D_3__Actinomycetales;D_4__Actinomycetaceae
D_0__Bacteria;D_1__Firmicutes;D_2__Clostridia;D_3__Clostridiales;__
D_0__Bacteria;D_1__Actinobacteria;D_2__Coriobacteriia;D_3__Coriobacteriales;__
D_0__Bacteria;D_1__Tenericutes;D_2__Mollicutes;D_3__Izimaplasmatales;D_4__gut metagenome
D_0__Bacteria;D_1__Firmicutes;D_2__Bacilli;D_3__Lactobacillales;D_4__Carnobacteriaceae
D_0__Bacteria;D_1__Actinobacteria;D_2__Coriobacteriia;D_3__Coriobacteriales;D_4__Coriobacteriales Incertae Sedis
D_0__Bacteria;D_1__Firmicutes;D_2__Clostridia;D_3__Clostridiales;D_4__Defluviitaleaceae
D_0__Bacteria;D_1__Cyanobacteria;D_2__Oxyphotobacteria;D_3__Chloroplast;__
D_0__Bacteria;D_1__Firmicutes;D_2__Bacilli;D_3__Lactobacillales;D_4__Enterococcaceae
D_0__Bacteria;D_1__Synergistetes;D_2__Synergistia;D_3__Synergistales;D_4__Synergistaceae
D_0__Bacteria;D_1__Actinobacteria;D_2__Actinobacteria;D_3__Micrococcales;D_4__Micrococcaceae
D_0__Bacteria;D_1__Tenericutes;D_2__Mollicutes;D_3__Mollicutes RF39;__
D_0__Bacteria;D_1__Actinobacteria;D_2__Coriobacteriia;D_3__Coriobacteriales;D_4__uncultured
D_0__Bacteria;D_1__Firmicutes;D_2__Clostridia;D_3__Clostridiales;D_4__Eubacteriaceae
D_0__Bacteria;D_1__Patescibacteria;D_2__Saccharimonadia;D_3__Saccharimonadales;D_4__Saccharimonadaceae
D_0__Bacteria;D_1__Lentisphaerae;D_2__Lentisphaeria;D_3__Victivallales;D_4__Victivallaceae
D_0__Bacteria;D_1__Firmicutes;D_2__Clostridia;D_3__Clostridiales;D_4__Family XI
D_0__Bacteria;D_1__Actinobacteria;D_2__Actinobacteria;D_3__Corynebacteriales;D_4__Corynebacteriaceae
D_0__Bacteria;D_1__Cyanobacteria;D_2__Melainabacteria;D_3__Gastranaerophilales;D_4__Candidatus Gastranaerophilales bacterium Zag_111
D_0__Bacteria;D_1__Firmicutes;D_2__Bacilli;D_3__Lactobacillales;D_4__Leuconostocaceae
D_0__Bacteria;D_1__Tenericutes;D_2__Mollicutes;D_3__Anaeroplasmatales;D_4__Anaeroplasmataceae
D_0__Bacteria;D_1__Cyanobacteria;D_2__Melainabacteria;D_3__Gastranaerophilales;D_4__Clostridium sp. CAG:306
D_0__Bacteria;D_1__Proteobacteria;D_2__Alphaproteobacteria;D_3__Rickettsiales;D_4__Mitochondria
D_0__Bacteria;D_1__Proteobacteria;D_2__Alphaproteobacteria;D_3__Caulobacterales;D_4__Caulobacteraceae
D_0__Bacteria;D_1__Firmicutes;D_2__Clostridia;D_3__Clostridiales;D_4__uncultured
D_0__Bacteria;D_1__Bacteroidetes;D_2__Bacteroidia;D_3__Bacteroidales;__
D_0__Bacteria;D_1__Bacteroidetes;D_2__Bacteroidia;D_3__Bacteroidales;D_4__Porphyromonadaceae
D_0__Bacteria;D_1__Firmicutes;D_2__Bacilli;D_3__Bacillales;D_4__Family XI
D_0__Bacteria;D_1__Tenericutes;D_2__Mollicutes;D_3__Mollicutes RF39;D_4__metagenome
D_0__Bacteria;D_1__Firmicutes;D_2__Clostridia;D_3__DTU014;D_4__uncultured bacterium
D_0__Bacteria;D_1__Proteobacteria;__;__;__
D_0__Bacteria;D_1__Tenericutes;D_2__Mollicutes;D_3__Mollicutes RF39;D_4__uncultured bacterium
D_0__Bacteria;D_1__Firmicutes;D_2__Bacilli;D_3__Bacillales;D_4__Staphylococcaceae
D_0__Bacteria;D_1__Actinobacteria;D_2__Actinobacteria;D_3__Propionibacteriales;D_4__Propionibacteriaceae
D_0__Bacteria;D_1__Proteobacteria;D_2__Gammaproteobacteria;D_3__Pseudomonadales;D_4__Pseudomonadaceae
D_0__Bacteria;D_1__Patescibacteria;D_2__Saccharimonadia;D_3__Saccharimonadales;__
D_0__Bacteria;D_1__Proteobacteria;D_2__Alphaproteobacteria;D_3__Azospirillales;D_4__Azospirillaceae
D_0__Bacteria;D_1__Proteobacteria;D_2__Deltaproteobacteria;D_3__Oligoflexales;D_4__0319-6G20
D_0__Bacteria;D_1__Proteobacteria;D_2__Gammaproteobacteria;D_3__Betaproteobacteriales;D_4__Neisseriaceae
Unassigned;__;__;__;__
D_0__Bacteria;D_1__Bacteroidetes;D_2__Bacteroidia;__;__
D_0__Bacteria;D_1__Firmicutes;D_2__Bacilli;D_3__Lactobacillales;D_4__Aerococcaceae

Figure S11. Bar plot showing the relative taxonomic frequency of the gut microbiota at the family level in males before, during and after the university examination period. Clinical Visit 1 (V1) occurred during the first half of the university semester, Visit 2 (V2) occurred during the university examination period, and Visit 3 (V3) occurred at the end of the university semester. Visit 1: control n = 3; walnut n = 5. Visit 2: control n = 3; walnut n = 2; Visit 3: control n = 3; walnut n = 2. Results from cohort 2 and 3 are pooled.

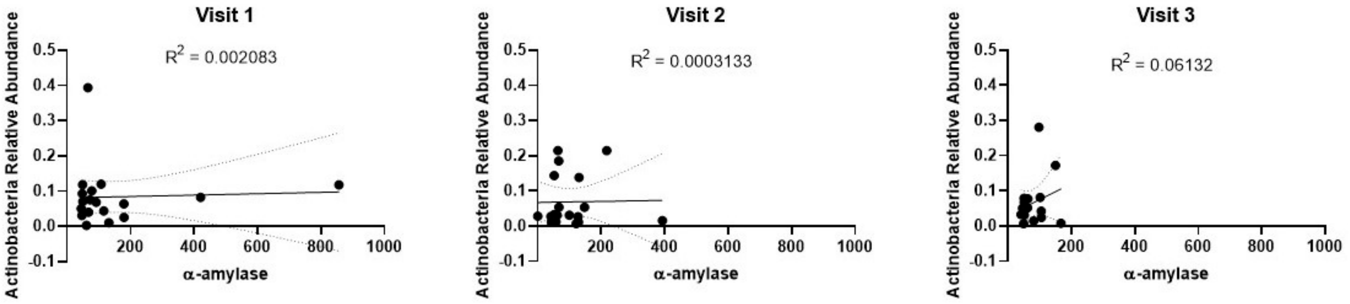

Figure S12. Correlations between Actinobacteria relative abundance and salivary α-amylase in females before, during and after the university examination period. Clinical Visit 1 occurred during the first half of the university semester, Visit 2 occurred during the university examination period, and Visit 3 occurred at the end of the university semester. Simple linear regression. Visit 1: control n = 14; walnut n = 11. Visit 2: control n = 11; walnut n = 10; Visit 3: control n = 11; walnut n = 9. Results from cohort 2 and 3 are pooled.

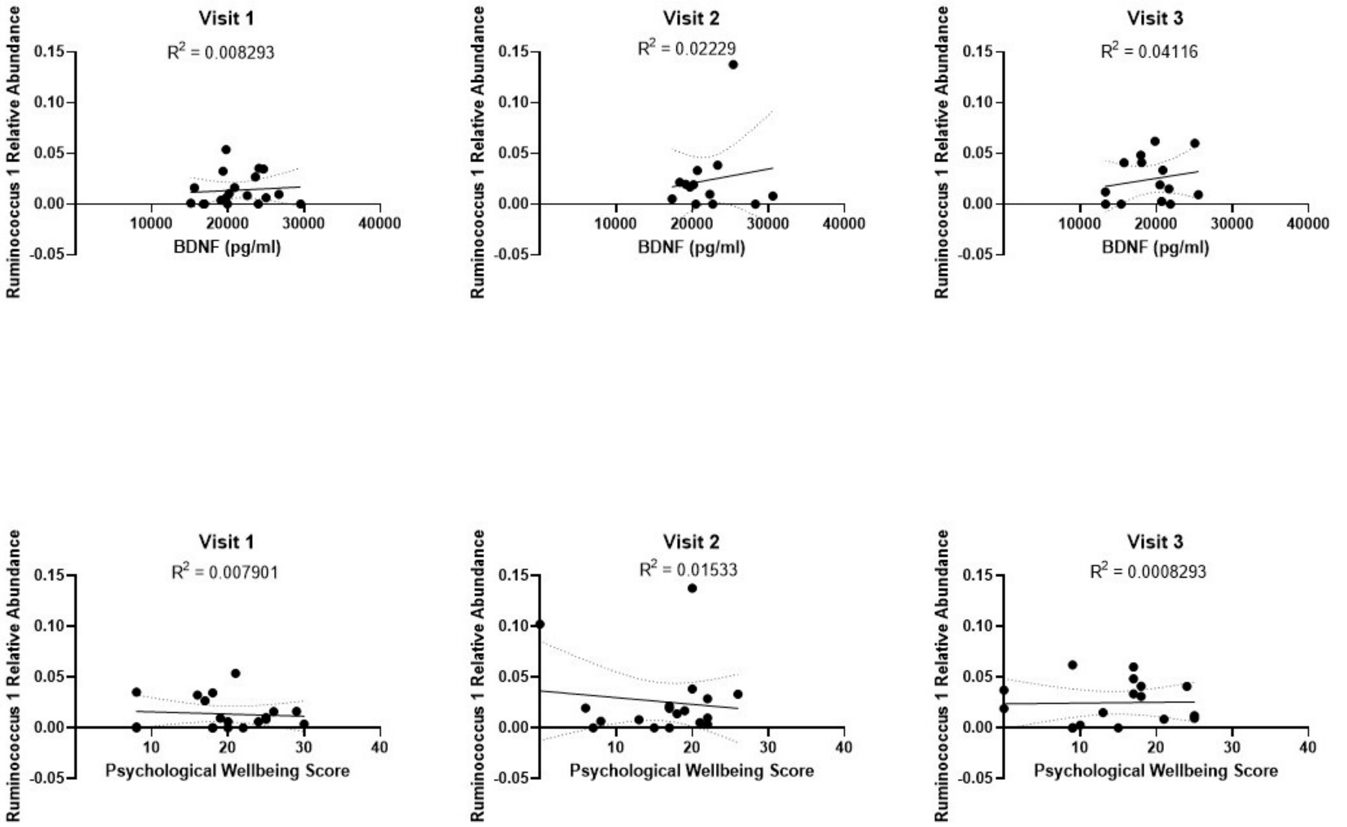

Figure S13. Correlations between *Ruminococcus 1* relative abundance, serum BDNF and Psychological Wellbeing Scores from the MHC in females before, during and after the university examination period. Clinical Visit 1 occurred during the first half of the university semester, Visit 2 occurred during the university examination period, and Visit 3 occurred at the end of the university semester. Simple linear regression. Visit 1: control n = 14; walnut n = 11. Visit 2: control n = 11; walnut n = 10; Visit 3: control n = 11; walnut n = 9. BDNF, mature brain-derived neurotrophic factor. MHC, Mental Health Continuum. Results from cohort 2 and 3 are pooled.
